# Extracellular Galectin-3 in Tumor Progression and Metastasis

**DOI:** 10.3389/fonc.2014.00138

**Published:** 2014-06-16

**Authors:** Anneliese Fortuna-Costa, Angélica M. Gomes, Eliene O. Kozlowski, Mariana P. Stelling, Mauro S. G. Pavão

**Affiliations:** ^1^Programa de Glicobiologia, Laboratório de Bioquímica e Biologia Celular de Glicoconjugados, Instituto de Bioquímica Médica Leopoldo de Meis, Hospital Universitário Clementino Fraga Filho, Universidade Federal do Rio de Janeiro, Rio de Janeiro, Brazil

**Keywords:** galectin-3, cancer, metastasis, glycosylation, Mgat5, lattices, tumor microenvironment

## Abstract

Galectin-3, the only chimera galectin found in vertebrates, is one of the best-studied galectins. It is expressed in several cell types and is involved in a broad range of physiological and pathological processes, such as cell adhesion, cell activation and chemoattraction, cell cycle, apoptosis, and cell growth and differentiation. However, this molecule raises special interest due to its role in regulating cancer cell activities. Galectin-3 has high affinity for β-1,6-*N*-acetylglucosamine branched glycans, which are formed by the action of the β1,6-*N*-acetylglucosaminyltransferase V (Mgat5). Mgat5-related changes in protein/lipid glycosylation on cell surface lead to alterations in the clustering of membrane proteins through lattice formation, resulting in functional advantages for tumor cells. Galectin-3 presence enhances migration and/or invasion of many tumors. Galectin-3-dependent clustering of integrins promotes ligand-induced integrin activation, leading to cell motility. Galectin-3 binding to mucin-1 increases transendothelial invasion, decreasing metastasis-free survival in an experimental metastasis model. Galectin-3 also affects endothelial cell behavior by regulating capillary tube formation. This lectin is found in the tumor stroma, suggesting a role for microenvironmental galectin-3 in tumor progression. Galectin-3 also seems to be involved in the recruitment of tumor-associated macrophages, possibly contributing to angiogenesis and tumor growth. This lectin can be a relevant factor in turning bone marrow in a sanctuary for leukemia cells, favoring resistance to therapy. Finally, galectin-3 seems to play a relevant role in orchestrating distinct cell events in tumor microenvironment and for this reason, it can be considered a target in tumor therapies. In conclusion, this review aims to describe the processes of tumor progression and metastasis involving extracellular galectin-3 and its expression and regulation.

## Introduction

**Figure 1:**
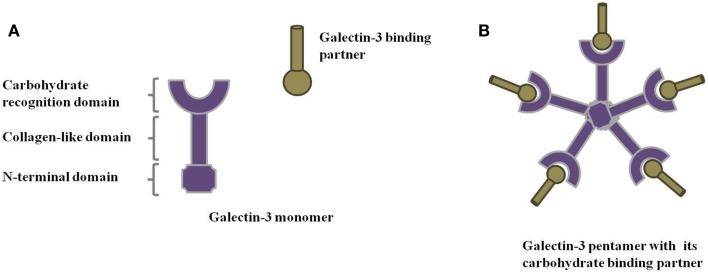
**Galectin-3 structure**. Schematic representation of **(A)** galectin-3 monomer and **(B)** galectin-3 oligomerization through its N-terminal domain in the presence of its binding partners.

Galectins comprise a family of animal lectins defined by their ability to recognize β-galactoside-containing glycoconjugates through a conserved carbohydrate-recognition domain (CRD). Based on molecular architecture, the 15 members of the galectin family are divided into 3 main groups: (1) prototype galectins, (2) tandem repeat galectins, and (3) chimera galectins. While prototype galectins are usually homodimers with two polypeptides containing a CRD each, tandem repeat galectins are monomers presenting two CRDs, connected by a linker region. Chimera galectins, in turn, consist of one CRD connected to a collagen-like sequence formed by Pro-Gly-Tyr tandem repeats and an N-terminal domain (Figure 1A). Some galectins are present in a variety of tissues, while others have a more specific localization. Galectins are involved in physiological and pathological events, such as cell proliferation and differentiation, apoptosis, immune response, cell differentiation, and tumor progression. The mechanisms underlying these aspects are currently under massive investigation.

Galectin-3, the only chimera galectin found in vertebrates, is one of the best-studied galectins. It is a versatile 29–35 kDa protein that participates in several biological processes: cell adhesion, cell activation and chemoattraction, cell growth and differentiation, cell cycle, and apoptosis (1–4). Galectin-3 occurs mainly in the cytosol, but can also transverse membranes reaching the nucleus (5) and mitochondria (6, 7). In general, galectins lack a signal peptide that would direct them to the classical secretory pathway; nevertheless, galectin-3 has been encountered in the extracellular environment. Once in the extracellular space, galectin-3 can interact with innumerous binding partners, mostly polylactosamine-rich molecules in the extracellular matrix (ECM) or on the cell surface, and plays key roles in the extracellular modulation of tumor progression (1, 8). The non-classical mechanism for galectin-3 secretion is not clear, but data obtained so far suggest that galectin-3 is secreted via exosomes (9) and its N-terminal domain is responsible for locating galectin-3 into these structures (10).

Despite galectin-3 expression in a variety of cell types and its involvement in several biological processes, this molecule raises special interest due to its remarkable role in regulating a broad range of cancer cell activities (11). Indeed, galectin-3 is often overexpressed in various human solid tumors and blood malignancies and, in many cases, this altered expression correlates with the stage of tumor progression, suggesting an influence of this molecule in disease outcome (2).

There are multiple lines of evidence pointing to the relevance of galectin-3 in malignant cell transformation (12, 13), tumor growth (14, 15), *anoikis* resistance (16), apoptosis inhibition (17, 18), angiogenesis (19, 20), cell adhesion (21, 22), cell motility (23), and cell invasion (24, 25). The last four are important steps of the metastatic process, in which extracellular, rather than intracellular galectin-3, plays a prominent role in both tumor cells and stromal cells present in the tumor microenvironment (19, 26–28).

In this review, we aim to examine recent data regarding the extracellular functions of galectin-3 in some steps of tumor progression and metastasis. We also discuss how cells at the tumor microenvironment may contribute to extracellular galectin-3 mediated tumor progression.

## Galectin-3 Binding Partners

### Galectin-3 has high affinity for β-1,6-*N*-acetylglucosamine branched glycans

Glycosylation is the most common post-translational modification occurring in proteins, with nearly half of all known proteins in eukaryotes being glycosylated. The presence of oligosaccharide moieties in proteins secreted or expressed on the cell surface affects protein conformation and localization. The structural complexity of oligosaccharides brings bio-specific information, mediating highly relevant molecular and cellular interactions. Indeed, changes in oligosaccharide structures are associated with many physiological and pathological events, such as cell growth (29), migration (30, 31), cell adhesion (32), endocytosis (33), transmembrane signaling (34, 35) autoimmunity, metabolic syndrome (36), and tumor development and metastasis (37, 38).

The tumor microenvironment is characterized by aberrant glycosylation, with oligosaccharide under- or overexpression and presence of novel carbohydrate moieties. These aberrations are mostly due to changes in the expression levels of glycosyltransferases in the Golgi compartment of cancer cells. Several evidences point to an increase in size and branching of N-linked glycans as a remarkable feature of colon and breast cancer (39, 40). This change is usually attributed to the increased activity or expression of β1,6-*N*-acetylglucosaminyltransferase V (Mgat5). This Golgi enzyme catalyzes the addition of β1,6-*N*-acetylglucosamine to both tetra-antennary N-linked and O-linked oligosaccharides, forming multi-antennary chains. These longer and more branched chains bind to galectins with higher affinity than less branched glycans (41, 42). Mgat5 is indeed responsible for the synthesis of preferred binding partners for galectins (2).

Galectin-3 has high affinity for β-1,6-*N*-acetylglucosamine branched glycans. Such interaction mediates binding of the lectin to many glycoproteins and glycolipids in the cell membrane, including carcinoembryonic antigen (CEA), mucin-1, lysosomal-membrane-associated glycoproteins (LAMPs)-1 and -2, Mac-1 and Mac-3, CD-98, CD-45, CD-71 (43–46), and the glycosylated transmembrane tyrosine kinase receptors for epidermal growth factor (EGF) (42, 47), transforming growth factor beta (TGF-β) (42), vascular endothelial growth factor (VEGF) (48), and others. Mgat5-related changes in protein/lipid glycosylation and density on cell surface lead to alterations in the clustering of those membrane proteins through lattice formation, resulting in functional advantages for tumor cells (see below). A list of the main galectin-3 binding partners is shown in Table 1.

**Table 1:** **Galectin-3 binding partners**.

Molecule	Reference
1. Carcinoembryonic antigen	(43–46)
2. Mucin-1	
3. Lysosomal-membrane-associated glycoproteins-1 and -2	
4. Mac-1 and -3	
5. CD-98	
6. CD-45	
7. CD-71	
8. Fibronectin	(49)
9. Collagen IV	(50)
10. Elastin	(51)
11. Laminin	(49)
12. Hensin	(49)
13. N-cadherin	(22)
14. Desmoglein	(52)
15. αvβ3 Integrin	(53)
16. VEGFR-2	(48)
17. NG2 (neuron-glial antigen 2) chondroitin sulfate proteoglycan	(54)
18. α3β1-Integrin	(54)

Increased β1-6 branching of other Mgat5 target proteins, such as cadherins, integrins, and other cytokine/growth factor receptors may enhance and promote tumor growth and metastasis (22, 23, 55). On the other hand, Mgat5 knockout (KO) mice have been shown to present suppressed polyomavirus middle T antigen-induced tumor growth and metastasis (56). Despite the low number of papers published on the direct effects of Mgat5 activity on galectin-3 function, both proteins are increased during tumor progression and deserve attention as suitable targets in malignant diseases.

### Lattice formation

Lattices are multivalent complexes of soluble galectins and glycoprotein receptors on the cell surface. These structures are important for organization of glycoprotein assemblies on the cell surface. Recent works have shown, through genetic and biochemical manipulation of glycosylation pathways or galectins themselves, that galectin–glycoprotein lattices affect the control of biological processes, including glycoprotein receptor turnover and endocytosis among other biological processes. Thereby, formation of galectin–glycan lattices may extend exposition of glycoproteins on the cell surface, affecting cell response to receptors ligands (57, 58).

**Figure 2:**
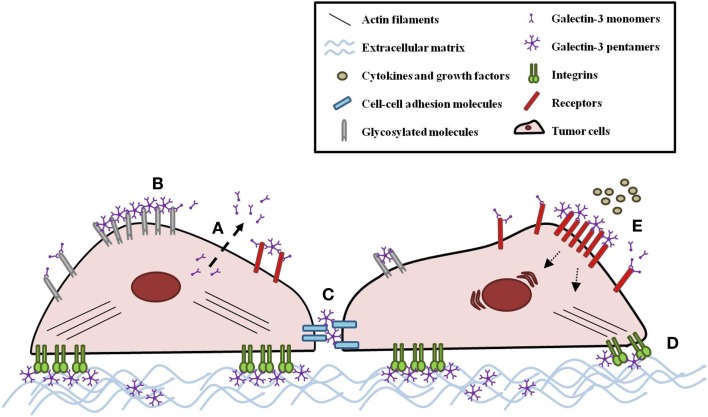
**Galectin-3 modulates tumor cell behavior**. **(A)** Galectin-3 monomers are secreted by a non-classical mechanism. Once in the extracellular space, galectin-3 monomers can interact with innumerous glycosylated molecules, such as receptors, cell–cell adhesion molecules, integrins, and ECM molecules. Upon monomeric galectin-3 interaction with a binding partner, additional galectin-3 monomers are linked to the complex through their N-terminal domain, establishing pentameric structures. **(B)** This complex cross-links carbohydrate-containing glycans, promoting the formation of organized galectin–glycan clusters termed lattices and modulating tumor cell behavior, such as **(C)** adhesion and **(D)** migration. **(E)** Galectin-3-glycan lattices also extend the exposition of receptors on the cell surface, affecting cell response to cytokines and growth factors.

Intracellular galectin-3 is mostly a monomeric soluble protein (59). Upon interaction of a monomeric galectin-3 with glycoproteins or glycolipids, additional galectin-3 monomers are linked to the complex through their N-terminal domain, establishing pentameric structures (Figure 1B) (1). This complex of multivalent interactions cross-links carbohydrate-containing glycoproteins or glycolipids, promoting the formation of organized galectin–glycan clusters termed lattices and modulating cell functions (Figure 2) (60, 61). Ultimately, galectin-3 helps to organize functional microdomains on the cell surface assuring proper transmission of extracellular signals into the cell (57, 58). It is worth emphasizing that the glycosylation pattern of galectin-3 binding partners is determinant for extracellular galectin-3 binding and lattice formation on the cell surface (33, 57).

## Extracellular Galectin-3 in the Invasion-Metastasis Cascade

During tumor progression, some cancer cells become capable of invading surrounding tissues, disseminating through blood or lymphatic vessels, penetrating basement membranes and endothelial walls, extravasating into parenchyma and colonizing distant organs (62). Although it is thought that only a few cells in the primary tumor are able to succeed in all these steps, this complex and dynamic process accounts for the majority of cancer-related deaths (63).

At the mechanistic level, invasion and metastasis processes involve changes in several classes of proteins engaged in cell–cell and cell–matrix adhesion, as well as in signaling pathways that control cytoskeletal dynamics (64). Extracellular galectin-3 seems to contribute to these processes and might have relevance in the invasion-metastasis cascade.

### Regulation of tumor invasiveness by extracellular galectin-3

The migratory and invasive activation of tumor cells is often associated with tumor progression (64). Galectin-3 expression was shown to increase migration and/or invasion of many tumor types, such as breast cancer (65), melanoma (14), lung cancer (66), sarcoma (67), gastric cancer (68), and chronic myelogenous leukemia (CML) *in vitro* (27). However, specific dependence on extracellular galectin-3 has not been determined in most of these studies.

The adhesive interaction with ECM binding partners is important for cell migration (64). In this regard, galectin-3 was shown to interact with ECM glycoproteins such as fibronectin, collagen IV, elastin, laminin, and hensin (49–51, 69). Moreover, exogenously added galectin-3 interacts with Mgat5-modified N-linked oligosaccharides on the surface of mammary carcinoma cells and stimulates α5β1-integrin activation, enhancing fibronectin fibrillogenesis and fibronectin-dependent tumor cell spreading, and motility (70). Therefore, galectin-3-dependent integrin clustering promotes integrin activation as well as ligand-induced integrin activation (70). Additionally, Src kinase-dependent caveolin-1 (Cav1) phosphorylation increases focal adhesion turnover, RhoA activation, and tumor cell migration in a galectin-3-dependent manner. Interestingly, galectin-3 and phosphorylated Cav1 (pCav1) act synergistically in this context, because overexpression of a non-phosphorylatable Cav1 mutant or disruption of galectin-3 lattice inhibit cell motility (71, 72). More recently, Boscher and Nabi showed that galectin-3 and pCav1-dependent integrin signaling mediate EGF stimulation of actin reorganization in circular dorsal ruffles, cell migration, and fibronectin remodeling. The authors proposed that Mgat5-dependent galectin-3 lattice enables EGF signal transduction that triggers galectin-3-dependent integrin activation. Then, the active integrin induces Src-dependent phosphorylation of Cav1 and RhoA/ROCK signaling (23).

*De novo* expression of galectin-3 in a galectin-null sarcoma cell line decreased cell adhesion to laminin-111 and promoted their migratory capacity on the same substratum, in a carbohydrate-dependent manner. Migratory modulation was associated with disassembly of stable focal adhesion plaque, as evaluated by the decrease of phosphorylated focal adhesion kinase (FAK) in lamellipodia of migrating cells and phospho-paxillin in focal complexes. The promigratory activity was also shown to be dependent on activation of PI-3 kinase pathway and AKT phosphorylation (67).

It is well established that loss of intercellular adhesions correlates with tumor invasion and metastasis, as it allows tumor cells to escape the primary tumor site. Indeed, during the migration process, intercellular junctions are actively remodeled (64). Recently, it was shown that extracellular galectin-3 can bind to N-cadherin in murine mammary cancer cells, and this association is mediated by Mgat5-generated *N*-glycans. More interestingly, galectin-3 binding to N-cadherin contributes to destabilization of cell–cell junctions by enhancing turnover of N-cadherin and other glycoconjugates, which might favor cell migration process (22). On the other hand, the binding of extracellular galectin-3 to *N*-glycans on desmoglein, a cadherin present on desmosomes, seems to promote the stability of cadherin at the cell surface and in epithelial intercellular adhesion on colon cancer cells (52). This extracellular galectin-3-mediated modulation of tumor cell motility deserves further investigation in different models.

### Extracellular galectin-3 in cell adhesion during tumor dissemination

The survival of tumor cells in blood or lymphatic circulation and their subsequent adhesion to vascular endothelium represent crucial steps for colonizing secondary sites (62). In this regard, exogenous or tumor-derived galectin-3 was shown to increase melanoma and colon cancer cells homotypic and heterotypic adhesions by interaction with oncofetal Thomsen–Friedenreich carbohydrate on the cancer-associated transmembrane mucin protein (MUC1) (21, 45, 73). Galectin-3-MUC1 interaction increases tumor cell aggregation and favors the formation of tumor micro-emboli, preventing *anoikis* initiation and enhancing circulating tumor cell survival (73). The mechanism responsible for this interaction seems to be MUC1 induction of cell surface polarization, exposing smaller adhesion molecules on the cell surface, including E-cadherin, CD44, and E-selectin ligands (73, 74).

Galectin-3–MUC1 interaction was shown to enhance heterotypic adhesion between tumor and endothelial cells under static and flow conditions (21, 45, 74, 75). Such interaction also increased transendothelial invasion and decreased metastasis-free survival in an animal model of experimental metastasis (74). In a xenograft model, galectin-3 was shown to be upregulated in breast tumor cells proximal to the stroma. This localized expression may facilitate tumor–stromal interactions and consequently improve endothelial cells adhesion, resulting in invasion and metastatic progression (76). High expression of galectin-3 was also observed in breast cancer patients, both on tumor cells and tumor–stromal cells of most specimens. Interestingly, the presence of galectin-3 in tumor-associated stroma was related to unfavorable prognosis. Nevertheless, the survival analysis disclosed no prognostic correlation to either cytoplasmic or nuclear localization of galectin-3 on breast tumor cells (77).

Another metastasis-promoting role of galectin-3 results from the crosstalk between circulating galectin-3 and endothelial cells. Through the use of cytokine array and human microvascular lung endothelial cells, it has been shown that high levels of galectin-3 (mimicking pathological concentrations) induce secretion of several cytokines, such as IL-6, G-CSF, and GM-CSF *in vitro* and *in vivo*. These cytokines enhance tumor cell adhesion to endothelial cells through upregulation of E-selectin, ICAM-1, VCAM-1, and integrin α_v_β_1_ on endothelial cells (78)_._

Radosavljevic et al. also addressed galectin-3 role in tumor metastasis. In this study, they showed that, compared with wild-type (WT) mice, galectin-3 KO mice presented a markedly reduced number and size of metastatic colonies in an experimental model of lung B16F1 murine melanoma metastasis. Taking into account *in vitro* data showing a lower binding of malignant cells onto lung tissue of galectin-3 KO mice, the authors suggested the involvement of stromal cell-derived galectin-3 on tumor cell adhesion to the metastatic target. Interestingly, NK cells maturation and anti-tumor cytotoxic activity were increased in galectin-3 KO mice. Therefore, galectin-3 host deficiency could impair the successful establishment of melanoma metastatic foci by decreasing the binding of melanoma cells onto target tissue and by enhancing NK-mediated anti-tumor response (28).

## Tumor Microenvironment Relevance for Extracellular Galectin-3 Functions

In the past decade many convincing observations proved that communication between cancer cells and the associated stroma plays a key role in driving tumor progression. These evidences placed a spotlight on the relevance of understanding the tumor microenvironment rather than focusing studies only on the biology of tumor cells (62, 79, 80). Tumor–stromal cells (e.g., endothelial cells, immune and inflammatory cells, cancer-associated fibroblasts, myofibroblasts, and mesenchymal stromal cells) behavior is affected by extracellular galectin-3 and these cells are also capable of secreting this molecule (19, 81–83). Despite these evidences, our understanding on the role of extracellular galectin-3 present at the tumor milieu is still limited.

## Extracellular Galectin-3 in Tumor Angiogenesis

The establishment of new capillary vessels is a vital process for continuous tumor growth and provides a pathway for dissemination of malignant cells (62, 84). Previous works had shown that human breast carcinoma cells expressing galectin-3, unlike its galectin-3 null parental cells, progressively grew and metastasized when inoculated into the mammary fat pad of athymic nude mice (85). Nangia-Makker and co-workers hypothesized the involvement of galectin-3 secreted by tumor cells in angiogenesis. In fact, the authors demonstrated that extracellular galectin-3, by means of its carbohydrate-recognition capacity, affects endothelial cell behavior regulating capillary tube formation *in vitro* and angiogenesis *in vivo* (19). Galectin-3 was required for the stabilization of epithelial–endothelial interaction networks and co-culture of these two cells types resulted in increased levels of secreted galectin-3. However, it was not investigated in this work whether the co-culture condition modulated galectin-3 secretion in epithelial, endothelial, or both cell types (76).

The molecular mechanisms for galectin-3 promotion of angiogenesis involve the binding of galectin-3 to α_v_β_3_ integrins on endothelial cells, inducing integrin clustering and activating signaling pathways that influence VEGF and basic fibroblast growth factor (bFGF) angiogenic activity, in addition to promoting FAK phosphorylation (53). Furthermore, the binding of galectin-3 to VEGFR-2 is enough to retain the receptor on the plasma membrane of endothelial cells and to generate VEGFR-2-clusters that boosts cell signaling effects (48). Additionally, neuron-glial antigen 2 (NG2), a transmembrane chondroitin sulfate proteoglycan expressed by pericytes in newly formed blood vessels, was also shown to induce endothelial cell motility and angiogenesis by forming the NG2–galectin-3–α3β1 integrin complex at the pericyte–endothelial cell interface (54).

Galectin-3 collagen-like domain is susceptible to matrix metalloproteinases cleavage (86). Galectin-3 cleaved form was found in the conditioned medium of epithelial–endothelial co-cultures (76). Compared to tumor cells harboring cleavable galectin-3, tumor cells transfected with cleavage resistant galectin-3 showed reduced tumor growth in athymic nude mice and diminished angiogenesis (87). A subsequent study indicated that cleaved galectin-3 is more efficient than intact galectin-3 in exerting chemotactic forces upon endothelial cells through upregulation of phosphorylated FAK, possibly leading to enhanced tumoral angiogenesis, due to recruitment of these cells to the tumor site (20). Interestingly, compared with intact galectin-3, cleaved galectin-3 presented increased binding to laminin (86) and endothelial cells (76). Therefore, the cleavage seems to improve galectin-3 affinity to binding partners. However, cleavage of the collagen-like domain is also thought to reduce galectin-3 ability to form dimers or higher order oligomers by self-association (86), suggesting that extracellular galectin-3 angiogenic effects may not be essentially dependent on lattice formation.

Considering that tumor-associated macrophages (TAM) are known to enhance local production of VEGF (88) and galectin-3 was shown to be necessary for optimal pro-tumor macrophage polarization (89), Machado et al. have used an interesting approach to study the role of galectin-3 in TAM-mediated tumor angiogenesis. Galectin-3 expression was reconstituted in a melanoma cell line that lacks this lectin and both expressing galectin-3 or galectin-3 null parental cells were inoculated in galectin-3 KO or WT mice. Interestingly, the absence of galectin-3 *per se*, either in melanoma cells or in the tumor stroma of galectin-3 KO mice, reduced tumor-associated angiogenesis. Furthermore, tumor microenvironment galectin-3 was shown to interfere with TAM recruitment, because infiltrating CD68^+^ cells were observed into the tumor mass, while these cells were only found in the periphery of galectin-3 negative tumors engrafted in KO mice. *In vitro*, galectin-3 KO mice bone marrow-derived macrophages (BMDM) showed a reduced basal VEGF secretion when compared with BMDM from WT mice, and were not responsive to VEGF secretion induction upon TGF-β1 stimulation. Besides, *in vitro* results also demonstrated that, even without any specific stimulus, galectin-3-expressing melanoma cells secrete larger amounts of VEGF than its galectin-3 null parental cells. Therefore, these *in vitro* data might explain the diminished angiogenesis observed *in vivo* when galectin-3 is absent in tumor cells and/or in the tumor stroma (26). This work reinforces the idea that extracellular galectin-3 plays a role in the organization of tumor microenvironment.

## Galectin-3 in Bone Marrow Microenvironment

Galectin-3 KO mice were first reported as viable and without any obvious physiological defects, suggesting that other members of the galectin family could exert galectin-3 functions (90). In contrast, subsequent works showed that these mice have deficiencies in immune/inflammatory cell accumulation and differentiation during experimental peritonitis (91–93) and in the course of *Schistosoma mansoni* infection (94, 95). Histological analysis of bone marrow from galectin-3 KO mice exhibited considerable modifications with reduced cell density and increased trabecular projections into the marrow cavity. Additionally, absence of galectin-3 reduced the levels of GM-CSF gene expression in bone marrow stromal cells (BMSC), increased the number of hematopoietic multipotent progenitors with the concomitant decreased capacity to differentiate into mature myeloid cell populations. In view of the fact that hematological malignancies are frequently associated with BMSC disorders that disturb the hematopoietic system, the authors propose the feasible involvement of galectin-3 in this context (95).

In consonance with the aforementioned supposition, Yamamoto-Sugitani and co-workers investigated whether galectin-3 would be involved in the intricate relationship that turns the bone marrow microenvironment into a sanctuary for leukemia cells by favoring resistance to therapy. In fact, *in vitro* results showed that galectin-3 expression in CML cells was increased by co-culture with BMSC. Moreover, galectin-3 overexpression in CML cells promoted proliferation, migration toward BMSC-derived soluble factors, and multidrug resistance. *In vivo* findings showed that galectin-3 overexpression on CML cells facilitates bone marrow homing and lodgment (27). Afterward, the group revealed that galectin-3 overexpression on CML cells promotes its paracrine growth by decreasing the action of the SERPINA1–albumin complex growth inhibitor, a serine protease inhibitor involved in various proteolytic processes on the cell surface and on the ECM (15).

## Concluding Remarks and Future Perspectives

As addressed in this review, countless evidences point to a relevant role of extracellular galectin-3 in crucial steps of tumor progression and metastasis. This lectin seems to orchestrate distinct events involving several cell types that constitute the tumor microenvironment. For this reason, galectin-3 has been explored as a target for cancer therapy. In this regard, a promising result was obtained with specific peptides against galectin-3 CRD that were capable of significantly inhibiting rolling and stable heterotypic adhesion of tumor cells to endothelial cells, as well as homotypic tumor cell aggregation (96). In another human melanoma mouse model study, it was found that N-terminal truncated galectin-3 treatment was effective in reducing tumor growth and metastasis to axillary lymph node, but not to lung and liver. This truncated galectin-3 form is thought to act as a dominant-negative inhibitor since it retains the ability to bind carbohydrates and competes with endogenous galectin-3 for carbohydrate binding sites. Considering that this truncated galectin-3 form lacks the ability to dimerize or oligomerize, galectin-3 would become unable to cross-link its binding partners on the cell surface and the ECM and to form lattice structures, thereby modulating tumor cell changes in terms of adhesion, signaling, motility, and invasion (97).

The prognostic value of galectin-3 expression in cancer patients is still a subject of discussion (76, 98). Nonetheless, galectin-3 has emerged as a useful parameter in diagnosis and/or prognosis of some malignancies (99–101). Extending the knowledge on the effects of extracellular galectin-3 on several cells that comprise the tumor microenvironment, as well as, extending the knowledge on galectin-3 specific functions in the different tumor types, will provide valuable support for diagnosis, prognosis, and cancer therapy advances. However, caution should be taken in extrapolating data from galectin-3 activity in *in vitro* and *in vivo* models to the clinical situation. In this regard, it will be pertinent and timely to further investigate galectin-3 expression in tissue sections of cancer patients and relate these findings to clinical history in order to determine the potential value of galectin-3 detection for routine histopathological application.

Considering that many of the studies with exogenous addition of galectin-3, aiming to explore its biological function, were performed with high concentrations of the protein, doubts could rise concerning the relevance of extracellular galectin-3 in all physiological and pathophysiological conditions mentioned here. However, considering that stromal cells seem to contribute directly to the pool of extracellular galectin-3, or indirectly through modulation of galectin-3 expression and/or secretion by tumor cells (27, 76, 82), it is reasonable to speculate that, in certain circumstances, the high concentrations of galectin-3 used *in vitro* might be achieved *in vivo*.

It is also important to mention that most galectin-3 studies were conducted using bi-dimensional culture models. However, the emergence of three-dimensional models for cell culturing in the last two decades allows the consideration of new aspects of cell behavior *in vitro*. Three-dimensional culture models allow cells to mimic their *in vivo* architecture with a more complex ECM arrangement, as well as a different distribution pattern of cell surface molecules (80, 102). Additional studies that take into account these changes in cell and environment architecture could be of great potential to reveal other putative binding partners, contributing to explain known functions or even unveiling new functions of galectin-3.

## Conflict of Interest Statement

The authors declare that the research was conducted in the absence of any commercial or financial relationships that could be construed as a potential conflict of interest.
